# Awareness, Knowledge, and Perceptions Regarding Rabies Prevention Among Rural Communities in Masaka District, Central Uganda: A Qualitative Study

**DOI:** 10.3389/fvets.2022.863526

**Published:** 2022-06-13

**Authors:** Clovice Kankya, Salome Dürr, Sonja Hartnack, Charlotte Warembourg, Justine Okello, James Muleme, Walter Okello, Tubihemukama Methodius, Grace Alobo, Terence Odoch

**Affiliations:** ^1^Department of Biosecurity, Ecosystems and Veterinary Public Health, School of Biosecurity, Biotechnical and Laboratory Sciences, College of Veterinary Medicine, Animal Resources and Biosecurity, Makerere University, Kampala, Uganda; ^2^Veterinary Public Health Institute, Vetsuisse Faculty, University of Bern, Bern, Switzerland; ^3^Section of Epidemiology, Vetsuisse Faculty, University of Zurich, Zurich, Switzerland; ^4^Department of Disease Control and Environmental Health, School of Public Health, College of Health Sciences, Makerere University, Kampala, Uganda

**Keywords:** dog bites, participatory epidemiology, Masaka district, key informant interview, focus group discussion, perception, rabies

## Abstract

Rabies is a zoonotic disease that is mainly transmitted to humans through dog bites. It remains a major public health threat in many Asian and African countries, including Uganda. The main objective of this study was to investigate awareness, knowledge, and perceptions of communities toward human and dog health related to rabies prevention, as well as dog management practices within Masaka district, central Uganda. Data collection involved nine key informant interviews (KIIs) and six focus group discussions (FGDs). Methods used during focus group discussions included qualitative interviews (using open-ended questions), simple ranking, and proportional piling. Data from KIIs and FGDs were analyzed using content analysis in NVivo (version 12.0). This study reveals that community members in the rural settings uses herbal concoctions in replacement or as an alternative to dog vaccination. Furthermore, the study reveals that dogs play the vital roles in the households like as they offer protection to people and household properties, despite being ranked second least among the household animals. The commonest livelihood activity was a small-scale mixed farming. Most of the households kept dogs, but they are ranked at second lowest in terms of economic value among all domestic animals. Free roaming and tethering were the common dog-keeping systems, and home-based feed (food leftovers, bones) was provided mainly to the tethered dogs. Rabies, also locally known as “*Eddalu Lyembwa”* (that can be translated as “madness of the dogs”), was ranked as the disease of most important among dogs, besides other common diseases such as skin diseases, venereal diseases, worm infestations, and tick infestations. Inadequate vaccination services for both humans and dogs were reported, and dog bite victims traveled a long distance to seek for post-exposure prophylaxis after dog bites. It can be concluded that there is a clear request for periodic mass vaccination campaigns against rabies among dogs, and access to vaccines within reasonable distances by humans after a rabies exposure, but also pre-emptive vaccination for those at high risk, such as veterinarians, needs to be improved.

## Introduction

Rabies, an acute viral infection of the central nervous system, is caused by the rabies virus (RABV), the first identified genotype of the Lyssaviruses, family Rhabdoviridae, and is transmitted *via* bites from infected mammals that excrete the virus through the saliva (1). Wild animals, such as bats, raccoons, skunks, and foxes, are the well-known rabies reservoir species worldwide (2). However, the domestic dogs (*Canis familiaris*) are by far the most important rabies reservoirs and vector for human rabies in the world, predominantly in African and Asian countries (3). Despite the joint efforts by the World Health Association (WHO), the World Organization for Animal Health (OIE), the Food and Agriculture Organization (FAO), and the Global Alliance of Rabies Control (GARC) to eliminate dog-mediated rabies from the human population worldwide by 2030, and the presence of highly effective vaccines making rabies fully preventable, the disease still causes an estimated global burden of 59,000 annual human deaths (4), leading to the eleventh rank in regard to mortality among all infectious diseases (1, 5).

In Uganda, an estimated 486 human rabies death occurs every year (6), and an average of ~13,900 human animal bites were registered annually from 2001 to 2015 in Uganda. Despite dogs being known to play the key roles for security and hunting within communities, the majority of the dogs in urban and rural areas are kept free roaming with increased interactions among dogs (7, 8). This free roaming behavior increases the risk for attracting infectious diseases, including rabies, from other species, either domestic or wildlife (9, 10). Furthermore, this dog management practice and irresponsible ownership might enhance persistence and wider spread of rabies within a local dog population as it involves greater movement of infected dogs (11). Although wide-scale dog mass vaccination is recommended as a key measure that will tremendously reduce canine dogs rabies and therefore also human exposure to the disease, routine vaccination of domestic dogs against rabies is not practiced in Uganda (12). Instead, sometimes, rabies-suspected dogs are killed indiscriminately, causing human moral distress by animal health-care providers and dog owners (7).

In Uganda, information on knowledge, attitude, and practices (KAPs) on rabies control was mainly investigated in health professionals, and KAP studies in the general public are limited (8, 13). Unfortunately, communities are not so much involved in the design of appropriate rabies prevention and control intervention strategies, which may result in low understanding and commitment to such measures. The use of qualitative epidemiology methods enables to capture the communities' perceptions and knowledge and plays a vital role in understanding the patterns of rabies in populations based on the communities' view (9). The aim of this study was to investigate community awareness, knowledge, and perception of the community members toward human and dog health related to rabies and dog management practices within Masaka district, Uganda, using a participatory approach. This paper contributes to the ending rabies a long-standing one health challenge to 0 by 2030.

## Materials and Methods

### Study Area and Study Sites

**Figure 1:**
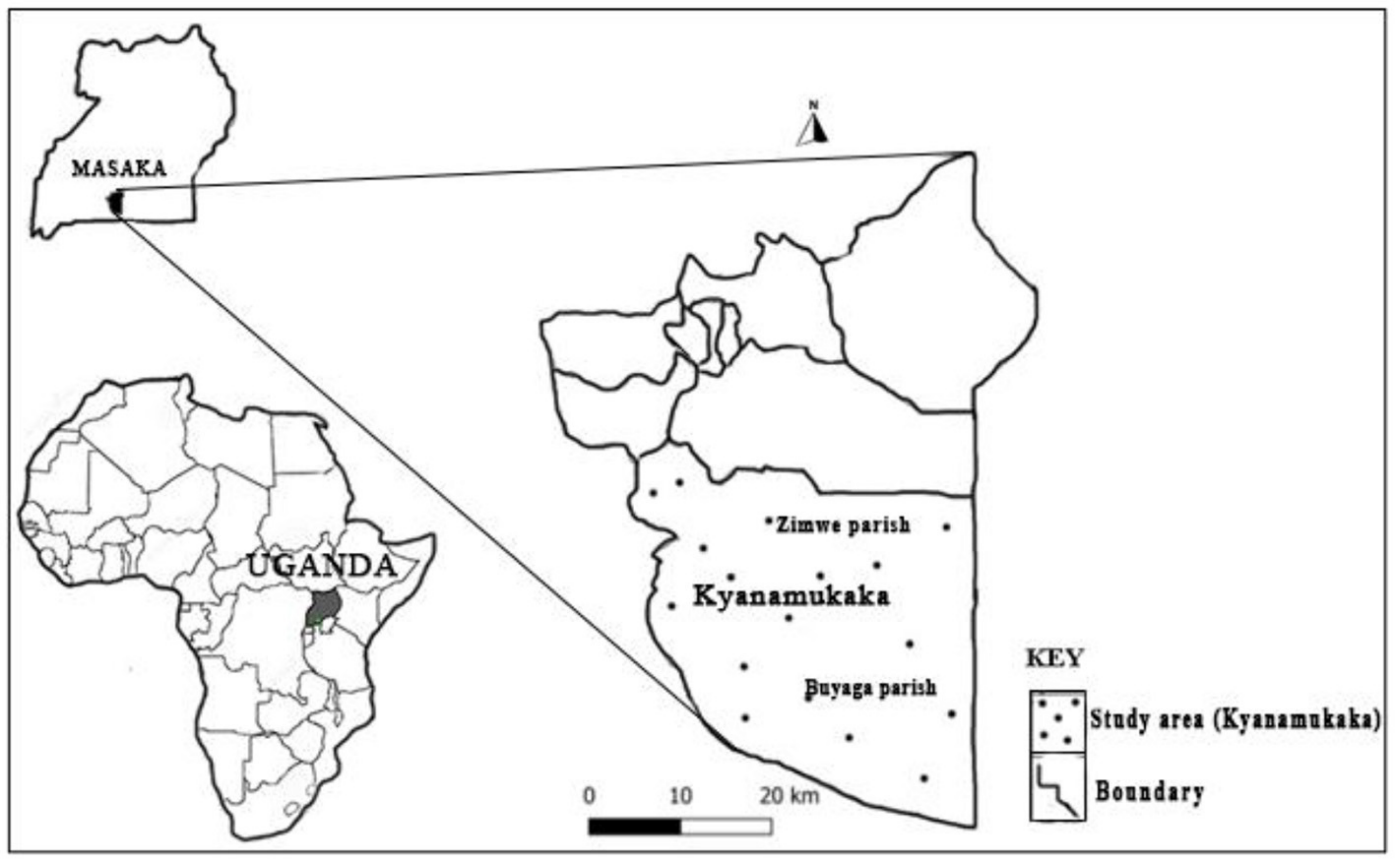
A map of Masaka district showing the study area (Kyanamukaka subcountry).

The study was undertaken as a part of a larger research project aiming to improve knowledge on domestic dog ecology and rabies control in Uganda (9, 14). The study was conducted in Kyanamukaka subcounty, which is a part of Masaka district in central Uganda (Figure 1), between May and July 2019. Masaka district covers an area of 1,298 km^2^ with a human population density of 253.4/km^2^. The town of Masaka, where the district headquarters are located, is approximately 140 km, by road, south-west of Kampala (Uganda's capital city) on the highway to Mbarara district. The average altitude of the district is 1,115 m above the sea level.

The study sites within Kyanamukaka were identified jointly with the district team that included the district veterinary officer, public health officer, and the community development officer during the pre-field visit. A total of three different study sites were selected and identified for the study, aiming to include sites with varying dog densities. The study sites consisted of two parishes of Zimwe (a rural parish) and Buyaga (a parish with small towns). In Buyaga, the following villages were included in the study: Buyaga, Diimu, Nkoma, Mpala, Kanyuga, and Bijunju, while in Zimwe parish, the following study villages were selected: Zimwe, Lukindu A and B, Kanamusabala, Katambi, Butaano, and Buna.

### Data Collection Methods

A number of two PE methods were applied: key informant interviews (KIIs) and focus group discussions (FGDs). These methods were applied according to the study by Bugeza et al. (15). The participants during the KIIs and the FGDs included both males and females. In addition, direct observations (dog breeds, dog management systems, dog health, and dog vaccination cards) were performed.

A total of nine KIIs were performed in three different villages (Buyaga, Diimu, and Zimwe) with the following persons: district veterinary officer, subcounty veterinary officer, subcounty chief, a community development worker, a primary school teacher, an environmental health officer, two village health team (VHT) member, and a parent whose child was a dog bite victim. A total of six FGDs, three per parish, were conducted. Each FGD comprising of 6–8 participants was conducted until saturation point was reached. A saturation point was reached when respondents were giving no new information anymore. Participants of the FGDs were mainly community members who own or used to own dogs, school teachers, local extension workers (animal and human health), and school children. For the FGDs, guided conversations on predetermined topics were used to structure the discussion.

The key informants and the community opinion leaders played a key role in deciding the appropriate venues and times for the KIIs and FGDs. These venues included, among others, school compounds and village public places. Convenient time for the interviews was agreed upon between the interviewee and the interviewer taking into account their routine work schedules. Interviews were conducted in the local language of the area “*Luganda,”* which the participants understood without need to interpret. An average of 30 min was utilized for KIIs and between 1 and 2 h for the FGDs.

All participants, both in the KIIs and in the FGDs, provided verbal consent, following reading out of the studies' aims and information related to their participation. The research protocol was reviewed by the research and ethical committees of the School of Biosecurity, Biotechnical and Laboratory Sciences, the School of Health Sciences of Makerere University, and it was presented to Uganda National Council of Science and Technology (approval number NS 640). Administrative clearance was sought from the district authorities. Participation in the study was voluntary, agreed on by verbal consent by the participants, and confidentiality was assured.

### Participatory Data Collection During KIIs and FGDs and Data Analysis

After an introduction of the study's background by the interviewer and FGD facilitator, respectively, the following information was collected: sociodemographic data on the interviewees, livelihood activities, dog ownership, and breeds of dogs kept. Next, the importance of dogs in different communities and challenges or constraints encountered while rearing dogs were discussed. The KIIs and FGDs also captured the local descriptions of the clinical presentation of common dog diseases. In addition, recommendations regarding dog health and proper management within the communities were discussed, and information on community knowledge and perceptions regarding dog bites was collected.

Simple ranking, as earlier described by Atuheire (16), was used during the FGDs to investigate variables such as the occurrence of dog breeds, economic importance of domestic animals, and common livelihood activities carried out in communities. A list of individual breeds of dogs was made by asking the participants to name dog breeds that are kept in the community. For each site, the breed names were written on cards and participants asked to agree on the order of the most frequent to the least kept dog breed. Similarly, a list of livelihood activities, of dog rearing methods, economic importance of animals, and dog management practices were generated. In addition, dog movement dynamics that including potential for interaction with the wild animals was assessed. Proportional pilling, as described by Williams et al. (17), was used to list and rank dog diseases according to their occurrence. A total of 100 pebbles were given to the participants. Participants distributed the pebbles representing counters to each square per severity of the dog disease in the community, with the largest pile of pebbles signifying the disease of highest severity and/or occurrence.

Audio-tapped data from KIIs and FGDs were first transcribed in English. The transcription process was quality controlled by well-trained qualitative data analysts. The research team then undertook a painstaking review process where each transcript was read thoroughly to identify the key statements that were repeated several times to identify the codes. This process was done by two independent reviewers, and the results were compared for triangulation by the third reviewer. Using NVivo (version 12.0), codes were developed from the identified topical sentences, and these were transferred to Microsoft Excel 2019. This guided the development of subthemes, which were later merged to form a total of five themes as stipulated in the Results section of this paper.

## Results

### Domestic Animals Kept and Dog Management Practices

Most participants from the FGDs reported that communities in Kyanamukaka kept a wide range of domestic animals including cattle, sheep, goats, poultry, rabbits, and dogs. Outcomes of the simple ranking revealed that dogs are very low in regard to their economic value as perceived by the informants, with only rabbits being ranked lower (Table 1). Still, it became evident in most of the KIIs and FGDs that almost all homes in the subcounty kept dogs at their homes. They added that dogs play a key role in protecting homes, crop gardens as well as for hunting purposes in the nearby bushes and forests. The FGDs through simple ranking revealed that the most common dog breeds kept in Kyanamukaka were local (indigenous dog) breeds.

**Table 1:** Simple ranking based on the economic importance of the domestic animals discussed among community members in Masaka district, Uganda.

**Livestock species**	**FGD in Study Site 1**	**FGD in Study Site 2**	**FGD in Study Site 3**	**FGD in Study Site 4**	**FGD in Study Site 5**	**FGD in Study Site 6**	**Total score**	**Rank**
Cattle	1	1	1	1	1	1	6	1
Poultry	2	3	5	3	2	2	17	2
Pigs	4	2	3	4	3	3	19	3
Goats	3	4	2	2	4	5	20	4
Dogs	5	5	4	5	5	4	28	5
Rabbits	6	6	6	6	6	6	36	6

*N.B: 1-high economic importance; 6- lowest economic importance; FGD, focus group discussion*.

*The common dog breeds kept in this study were the local breeds*.

The results from the KIIs indicated that dogs are observed to interact with several wild animals such as hares, squirrels, jackals, and antelopes. This interaction is greatly influenced by the dog rearing method. It was found that two main dog rearing methods exist, i.e., free roaming and tethering. The free roaming method applies to two categories of dogs, i.e., (a) those affiliated to households (left to move freely in the village, trading centers, schools, garbage points, and they return to the households at their convenience); (b) those that are not affiliated to any owner nor household, but freely roam within the community. It is imperative to note that some participants referred to stray dogs while talking about free roaming dogs. The latter type of dogs scavenges for their feed, and there is no special form of attention (veterinary care and feed supplements) given to such dogs.

Under the tethering rearing method, it was noted that dogs are either confined in a cage or chained to a stationary point where they are fed throughout the day. These dogs are mainly owned by men in a household even though women and children play a critical role in their care and feeding. In this rearing method, the dogs are released at night to provide security and protection of the owner and his/her property. Some key informants, especially from the urban part of the subcounty, affirmed that the owners of the tethered dogs provided them with feeds such as potatoes, maize, posho, water, bones, or leftovers from restaurants. Also, veterinary care was typically provided to these dogs.

“*In event that the dog falls sick, the veterinary officer or the community veterinary assistant is called upon to offer diagnostic and treatment services to the affected dog and* s*uch dogs are also vaccinated against rabies and other dog disease*”, veterinary assistant from Buyaga parish reported.

It is important to note that the rearing method also influences the dog population dynamics or turnover, which varies by various ways within a community.

“*On average, a female local dog breed gives birth to approximately 6-9 puppies which is a large number to be kept at home due to limited space, feed and food. In most cases, we either give away to homes/ individuals that want them or and we throw away the remaining puppies in areas where people are interested and can pick them”*, stated in the FGD in study sites 3 and 6.

### Livelihood Activities and Their Impact on Dog Management

Livelihood activities support the generation of food for humans, feed for animals (including dogs), and income of the households. The majority of the FGD participants from Zimwe parish recognized that such income is normally utilized to cater for household needs, including costs associated with rearing of dogs and their veterinary health care. However, it is imperative to note that those in Buyaga parish utilize their income by investing in business, such as trading. Veterinary officials from Kyanamukaka subcounty reported that these livelihood activities greatly affect the dog management practices, which in turn influence dog keeping practices as presented above.

From all the KIIs, it was said that the most common livelihood activity was farming from which livestock, and food crops were produced on a relatively subsistence or small-scale production levels. The integration of crop faming and livestock rearing (mixed farming) was ranked as the topmost activity. Food crops such as maize, beans, sweet potatoes, cassava, and banana plantations and cash crops such as coffee and eucalyptus trees were reported to be grown in the study area. The food crops from the gardens greatly contributed to feed for the dogs in the community. During the FGDs, it was revealed that females were more involved in crop farming whereas males were more involved in livestock rearing. In addition, dogs eat dead animals (domestic and wild) that could not be consumed by humans. Some FGD participants thought that such human condemned meats or dead animals could be a source of dog diseases and infections.

### Community Awareness of Common Dog Diseases

**Figure 2:**
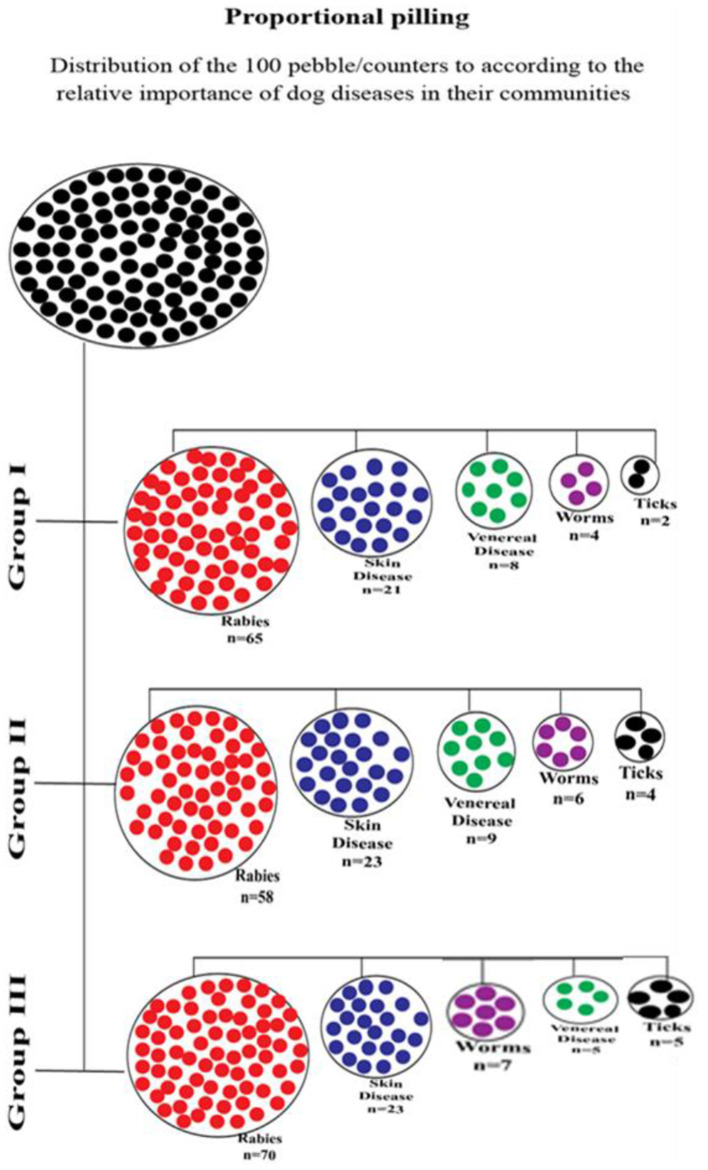
Proportion pilling results on dog diseases from three focus group discussions conducted in Masaka district, Uganda.

During the FGDs, several dog diseases and their respective local names were listed (Table 2). These were then subjected to proportional piling, where rabies was ranked highest from all the dog diseases listed (Figure 2). In addition, common signs and symptoms were noted for each of the listed diseases and these supported the ranking process. FGD participants also ranked a number of skin diseases of dogs as important, which ranged from fungal, parasitic (such as scabies), and viral diseases. Information from KIIs and FGDs revealed that communities were aware that some of these common dog diseases and infections are transmissible to humans mainly through dog bites. The strong perception of the community that diseases are transmitted from dogs to humans *via* bites highlights the prominent public health significance of rabies in the population.

**Table 2:** Signs, symptoms, and ranking of common dog diseases investigated by focus group discussions in Masaka district, Uganda.

**Disease/disease complex**	**Local name**	**Common clinical signs and symptoms**	**Rank**
Rabies	*Eddalu Lyembwa*	The dog is mad, increased saliva, very aggressive; fear of water, attacks every one including the owner. It eats anything including non-living objects such as sticks and stones	1
Skin Diseases	*Olukuku lwembwa*	Itching, irritation, scratching, loss of hair, skin swellings, thickening of the skin	2
Venereal Diseases	*Ebijaga*	Swollen genital organs, severe irritation on the genital area, sometimes discharges, restlessness	3
Worms	*Enjoka*	Worms and segments in dog stool, loss of body condition, dogs tend to eat too much	4
Tick Infestation	*Enkwa*	Visible ticks of the body parts, ear, eyes, thigh, arms, loss of condition	5

### Community Knowledge and Perceptions on Dog Bites

Most of the FGD participants in both parishes revealed that dog bites are very common among children while executing their domestic activities such as fetching water and collecting firewood. In addition, the FGD in Buyaga parish indicated that dog bites were one of the big threats that affect their pupils on their way to and from school. It is important to note that communities recognize that dog bites affect the general population and not only people keeping livestock such as goats, pigs, sheep, and rabbits.

“*Dogs whether chained or not, whether freely moving without owner or owned can attack people especially our children and our animals. We are all concerned and I remember we sometime contacted the veterinary office at the sub-county but they did not kill these dogs because some had their owners. These dogs like biting small animals like chicken, rabbits, ducks, goats, sheep and sometimes calves”*, reported by FGD in Buyaga village.

From the findings, it was clear that community members were alert and conscious of any dog bite, as they perceived every biting dog to be rabid.

“*It is difficult to know at community level which biting dog is rabid or not, therefore, it is necessary that all bites are suspected to come from a rabies positive dog until further investigations are conducted to confirm otherwise. This is why majority of our farmers get worried whenever a dog bites a human or their animals. In fact, even us as veterinarians need to be vaccinated to be safe”*, said by a veterinary assistant in Diimu village, Buyaga parish.

Furthermore, FGDs revealed that manifestation of rabies in dogs included signs such as extensive barking of dogs, increased salivation, restlessness, madness, and the dog appears in a choked state. It was also highlighted that the signs in humans included widening of the eyes, increased salivation, lacrimation (crying), and restlessness.

“*In our communities here, dog bites normally happen when mad-like dogs attack people who start throwing stones, sticks, logs on to a mad dog. This in turn triggers the restless and mad dog to chase people and consequently biting them. They also tell us that once a dog with rabies bites a human being, they start barking like dogs and later die. But we have not seen a human rabies case and that is why we fear the dog bites so much”*, reported during the FGD in Nkoma village, Buyaga parish.

### Management of Dog Bites and Prevention of Rabies

The findings of the FGD and KII revealed that in an event of a dog bite, the victim is always rushed to the nearby health facility supported by relatives or neighbors using a bicycle or a motorcycle. It was however noted that some of the health facilities within the communities do not have the vaccines due to their complex storage requirements (cold chain). Evidence was picked from a KII with a parent of a dog bites victim in Mpala village, Buyaga parish:

“*Toward the Christmas of 2018, my last born was bitten by a dog while he was coming back from the borehole in the evening. Community members rushed him to the nearest health center II and there were no drugs to help my son. The ‘doctors' at the health center II referred us to Masaka Regional Referral Hospital where we finally got the treatment and my son was saved”*.

In all the FGDs, participants indicated that the victims of dog bites were referred to Masaka Regional Referral hospital, which is approximately 20 km from Kyanamukaka subcounty for post-exposure prophylaxis. It is imperative to note that some dog bite victims who could not be managed from Masaka Regional Referral Hospital were referred to hospitals in Kampala and Wakiso districts, which are as far as around 130 km away. One of the key informant interviews with a Village Health Team member lamented that:

“*Our people travel long distances to access the healthcare services. The good thing is that services are offered in cases of dog bites.”*

Communities have embraced local concoctions from herbs in the prevention and control of rabies in humans and dogs. One of the commonly used herbs was called *Dimiryambwa*, which is a local concoction administered to dogs in the dosage equivalent to size of two tea spoons for the prevention of infecting humans. This is often provided to dogs by communities as an alternative to dog vaccination against rabies.

During one of the key informant interviews, it was reported that

“*Dogs often go to the nearby bushes and eat or feed on star grass (Dimiryambwa) and when they return, you notice them defecate and vomit the grass.”*

In case of a dog bite, the local communities use a commonly known local herb called “*Olucwamba,”* which is tied around the dog bite wound as a form of first aid.

In addition, from all the KIIs and FGDs, there was an outcry that the rabies treatment and dog vaccination services should be brought nearer to the community.

“*Some people want to vaccinate their dogs but they do not know where to find these services. We are all concerned because most of our dogs are not vaccinated, which puts our people in this community at risk of catching rabies. Yet the treatment for human rabies cases is also hard to get”* said in the FGD in Butaano village, Zimwe parish.

## Discussion

Rural communities such as those in Kyanamukaka subcounty, Masaka district practice a wide range of livelihood activities to support continuity of life (18, 19). In this study, results indicate that communities practice both livestock rearing (sheep, goats, cows, and poultry, among others) and crop farming (maize, beans, sweet potatoes, cassava, and banana plantation). However, wild animals and thieves negatively affect production and productivity and thus threatening these ventures (18). Therefore, the large majority of people in these communities tend to rear dogs as a major source of security for the protection of livestock, crops, and themselves. Products and by-products from crops (food, leftovers) and livestock (eggs, meat, milk, bones) enterprises provide feed for the dogs kept. Similar findings highlighted of dogs being a critical source of security and hunting in Uganda and South Africa, respectively, were highlighted by other studies (4, 5). However, it is noteworthy that the dogs were ranked as the second last important domestic species in regard to the economic value and only before rabbits. This may be due to dogs being a non-edible species in Masaka district, Uganda.

Dogs are kept under homebased tethering (chaining or in a cage) and free roaming rearing methods (19). In this study, it was noted that there are specific dogs that do not belong to any of these two major systems and are referred to as “stray dogs.” Dogs that are not under anyone's management or ownership freely mingle with the community dogs, posing a risk to animal and public health (20). Indeed, all free roaming dogs pose a potential risk for disease transmission (in case of non-vaccination status) or other harm, even if they just roam during the night. In addition, as noted in another study (21), dogs especially in rural communities, if not tethered, interface with wild animals, which increased the risk of disease transmission, including rabies, in both directions (22). Therefore, there is a dire need for improved management for dog movement within different ecosystems to minimize the risk of dog-related disease transmission to animals and humans.

Our study revealed that rabies is the dog disease perceived most important and feared in Masaka district, among dog diseases. Despite this, communities mentioned other dog diseases such as skin diseases, venereal diseases, worm infestations, and tick infestations as being also important. These findings defer from the studies by Amuguni et al. (23) and Aiyendun et al. (24), which considered leptospirosis and parvovirus as the most important dog diseases.

Interestingly, our study also found that community members were aware that rabies is associated with the dog bites. This was evidenced by their recognition of the clinical signs of rabies as madness, increased salivation, aggressiveness, uncontrolled attacks on humans, including the owner. Such levels of awareness on community are paramount for community-initiated strategies to control dog bites, thus minimizing rabies transmission risk to humans (23).

This study found that there are limited vaccination services recognized by the communities within the district, which poses a big threat to human and animal health. Prevention and control of dog-related diseases such as rabies is fundamental in dog management. Results from this study reveal that communities utilize both traditional and modern methods for wound healing among humans and rabies-like symptoms among dogs. Such findings emphasize the burden associated with dog bite and challenges of rabies management in rural communities. Other studies from Tanzania and Uganda report similar findings regarding the use of indigenous methods in the treatment and control of dog bites and rabies (24, 25).

Results from this study reveal a high number of free roaming unvaccinated dogs, associated with free roaming dog rearing systems, and a low value attributed to dogs compared with other domestic animals kept. This could be attributed to the fact that general dogs care, which include among other vaccinations, is often less prioritized in the rural communities. The limited care to most dogs thus results into reduced magnitude of connection between the dogs and the community member perceived as having less value.

Rabies is still a problem in the Masaka district's rural populations, and it has continued to harm them; however, their levels of understanding are improving as a result of prolonged exposure to dog bites. Despite the availability of local concoctions, communities have sought modern post-exposure prophylaxis from nearby health facilities in cases of dog bites. This is, however, hampered by the long distances and the unavailability of the required vaccines and technical expertise to conduct the vaccination. Such findings are common in most rural settings of developing countries where the health-care system is crippled by several infectious diseases neglecting others such as rabies (26). It is noteworthy that the switch to alternative, mostly indigenous traditional methods, is triggered by the lack of vaccines available for both humans and dogs. The limited resources in management and treatment of rabies and dog bites are also a huge challenge undermining the reduction of rabies cases across the country. Investment into dog vaccination may not only be the most effective way to control rabies, but may also increase the perceived economic value of the dogs kept, which in turn might motivate dog owners to invest into better management practices, such as providing sufficient and suitable feed and shelter.

In this study, we acknowledge that being qualitative in nature, the participants during the KIIs and the FGDs could have possibly given their view toward the study subject and not that of the general rural populations. However, th authors are certainly confident that this probable bias was appropriately managed at the different stages of the data analysis.

## Conclusion

In this study, we employed qualitative tools to investigate community awareness, knowledge, perception, and practices on dog management, dog health, and human dog bites within a rabies endemic area of Masaka district. Results from this study reveal a high number of free roaming unvaccinated dogs, associated with free roaming dog rearing systems, and a low value attributed to dogs compared with other domestic animals kept. In addition, rabies was ranked highest among all canine disease, and dog bites were reported as the greatest risk in regard to dog-related issues. This was linked to the limited availability of post-exposure prophylaxis of bite victims and the lack of extension of health services in remote sites, the use of traditional medicine to treat dog bites, and poor dog management practices within the district.

This study further highlights the communities' request for a concerted effort of multiple stakeholders in human and animal health units, departments, and agencies in the struggle to end dog bites and rabies in low resource districts of Uganda. There is a clear request from the communities for periodic mass vaccination campaigns against rabies among dogs, and also high-risk humans, such veterinarians, should be considered for preventive vaccination. Sensitization and outreach service activities addressing dog owners and general community about proper dog management practices and health-seeking behaviors in case of dog bites have been found to potentially greatly reduce the burden of rabies in the country.

## Data Availability Statement

The raw data supporting the conclusions of this article will be made available by the authors, without undue reservation.

## Ethics Statement

The studies involving human participants were reviewed and approved by Makerere University School of Biosecurity, Biotechnical and Laboratory Sciences Research Ethics Committee. The patients/participants provided their written informed consent to participate in this study.

## Author Contributions

CK, TO, and SD: conceptualization. WO, GA, JO, and JM: data collection. CK, JO, TM, and JM: formal analysis. CK, TO, SD, SH, and CW: investigations. JO, CK, JM, and TO: methodology. TO, CK, and JM: discussion. CK, JO, TO, SH, CW, and SD: writing original draft. CK, JO, TO, JM, SD, SH, and CW: writing final draft. SD, SH, and TO: resources. CK, SD, SH, CW, and JM: supervision. All authors contributed to the article and approved the submitted version.

## Funding

The funds utilized in this study were from the Wolfermann-Nägeli Foundation (Grant 2028/28), Hanela Foundation, Switzerland, and Makerere University through the dog ecology project.

## Conflict of Interest

The authors declare that the research was conducted in the absence of any commercial or financial relationships that could be construed as a potential conflict of interest.

## Publisher's Note

All claims expressed in this article are solely those of the authors and do not necessarily represent those of their affiliated organizations, or those of the publisher, the editors and the reviewers. Any product that may be evaluated in this article, or claim that may be made by its manufacturer, is not guaranteed or endorsed by the publisher.

## Abbreviations

FAO: Food and Agriculture Organization
FGD: Focus group discussion
GARC: Global Alliance of Rabies Control
KAP, Knowledge, attitude: and practices
KII: Key informant interview
OIE: World Organization for Animal Health
RABV: Rabies virus
WHO: World Health Organization.
